# Age-Adjusted PSA Levels in Prostate Cancer Prediction: Updated Results of the Tyrol Prostate Cancer Early Detection Program

**DOI:** 10.1371/journal.pone.0134134

**Published:** 2015-07-28

**Authors:** Isabel Heidegger, Josef Fritz, Helmut Klocker, Renate Pichler, Jasmin Bektic, Wolfgang Horninger

**Affiliations:** 1 Department of Urology, Medical University Innsbruck, Innsbruck, Austria; 2 Department of Medical Statistics, Informatics and Health Economics, Medical University Innsbruck, Innsbruck, Austria; 3 Department of Urology, Division of Experimental Urology, Medical University Innsbruck, Innsbruck, Austria; Cedars Sinai Medical Center, UNITED STATES

## Abstract

**Objective:**

To reduce the number of unnecessary biopsies in patients with benign prostatic disease, however, without missing significant PCa the present study re-evaluates the age-dependent PSA cut-offs in the Tyrol Prostate Cancer (PCa) early detection program.

**Patients and Methods:**

The study population included 2225 patients who underwent prostate biopsy due to elevated PSA levels at our department. We divided our patient collective into four age groups: ≤49 years (n = 178), 50-59 years (n = 597), 60-69 years (n = 962) and ≥70 years (n = 488). We simulated different scenarios for PSA cut-off values between 1.25 and 6 ng/mL and fPSA% between 15 and 21% for all four age groups and calculated sensitivity, specificity, confidence intervals and predictive values.

**Results:**

PCa was detected in 1218 men (54.7%). We found that in combination with free PSA ≤21% the following PSA cut-offs had the best cancer specificity: 1.75 ng/ml for men ≤49 years and 50-59 years, 2.25 ng/ml for men aged 60-69 years and 3.25 ng/ml for men ≥70 years. Using these adjusted PSA cut-off values all significant tumors are recognized in all age groups, yet the number of biopsies is reduced. Overall, one biopsy is avoided in 13 to 14 men (number needed to screen = 13.3, reduction of biopsies = 7.5%) when decision regarding biopsy is done according to the “new” cut-off values instead of the “old” ones. For the different age groups the number needed to screen to avoid one biopsy varied between 9.2 (≤49 years) and 17.4 (50-59 years).

**Conclusion:**

With “new”, fine-tuned PSA cut-offs we detect all relevant PCa with a significant reduction of biopsies compared to the “old” cut-off values. Optimization of age-specific PSA cut-offs is one step towards a smarter strategy in the Tyrol PCa Early Detection Program.

## Introduction

The use of prostate-specific antigen (PSA) for early detection of prostate cancer (PCa) has been shown to contribute to a favorable shift in tumor-stage at diagnosis resulting in decreased PCa morbidity and mortality [1,2]. However, PCa screening is one of the most controversial topics in the field of urology as a high number of PCa detected by PSA screening are low risk cancers which probably never become aggressive and thus lethally during patients´ life [3].

In general, PSA is a kallikrein-like serine protease with low chymotrypsin-like enzymatic activity produced by the epithelial cells of all types of prostatic tissue [4]. In 1986, PSA was approved by the USA Food and Drug Administration (FDA) to monitor cancer relapse after curative therapy and since the early nineties PSA is used as a tool for detecting PCa. However, the most important limitation of PSA is its specificity: as PSA is organ-specific it can be elevated not only by cancer of the prostatic gland but also in non-malignant conditions such as benign prostatic hypertrophy and prostatitis [5]. Moreover, PSA levels are highly variable over time and can be affected by manipulations of the prostate [5].

The rationale for PSA screening is the potential to reduce the risk of PCa morbidity and death through early detection. However, it is uncertain whether the benefits associated with PSA testing are worth the harms associated with screening and thus overtreatment of the disease. Randomized controlled studies provided disaccording results regarding real reduction in mortality through PSA screening, while all agreed on the risks of overdiagnosis and overtreatment: The European Randomized study of Prostate Cancer (ERSPC) study has shown a 21% reduction of PCa mortality after 11 years of follow up in screened patients [6]. Also their recently published 13 years follow-up update including 182160 men aged between 50 and 74 years confirmed that mortality was significantly lower in the screened group compared to the control group [7]. As a sub-analysis of the ERSPC trial, the Göteborg PCa screening trial reported that PCa mortality was reduced almost by 50% over 14 years through PSA at median age of 56 years and screening intervals of two years [8]. However, the American PSA screening trial (PLCO trial) assigning 76693 patients found that PCa mortality rates did not significantly differ between screen-detected individuals and the control group after a follow up of 7 years [9]. Comparing both studies, it has to be considered that in the ERSPC study men were much younger (median age: 56 year vs. >60 year in the PLCO trial), and the screening intervals were two years (PLCO study 4 years). Moreover, the ERSPC has a longer follow up compared to the PLCO trial.

Based on this data, the current EAU guidelines do not recommend widespread mass screening for PCa, however they recommend a baseline PSA determination at age 40 [5]. In contrast, the guidelines of the American Society of Urology (AUA) do not recommend routine screening for average-risk men ≤ 55 years [10]. Furthermore, according to the EAU guidelines, PSA testing is not necessary in men >75 years and with a baseline PSA of 3 of ng/ml because of their very low risk of dying from PCa.

In Tyrol, a state in Austria with a current male population of 353.910 *(01*.*01*.*2014*, *Statistics Austria)*, PSA testing was introduced in 1988, and since 1993 it has been offered cost free in an organized form to men aged between 45 and 74 years [11,12]. Analyzing long term survival rates we were able to show a significant reduction in PCa mortality with a risk ratio of 0.70 (95% confidence interval 0.57, 0.87) for Tyrol, compared to the mortality rate in the period from 1989 to 1993 [13]. The idea behind annual screening is the consideration that annual PSA measurement might detect PCa at early, organ confined and thus curable stages [14].

The present study re-evaluates the age-dependent PSA cut-offs used since 1995 in our early detection program, with the aim to reduce the number of unnecessary biopsies in patients with benign disease, however, without missing PCa, in particular without missing significant PCa [11,12].

### Patients and Methods

The present study was approved by the ethical committee of Medical University Innsbruck (UN3174, AM 3174). Written informed consent to participate in research studies was obtained from all patients.

### Study protocol and population

Patients who were assigned by an external physician were excluded from the study due to lack of complete data as well as to avoid heterogeneous data acquisition.

We included 2225 Caucasian patients who underwent prostate biopsy at our department between 1995 and 2012 and of whom all data were completely documented in our biobank. Age adjusted PSA levels used between 1995 and 2012 (49 years: ≥1.25 ng/mL, 50–59 years: ≥1.75 ng/mL, 60–69 years: ≥2.25 ng/mL, ≥70 years: ≥3.25 ng/mL), together with percentage-free PSA levels of <18% (fPSA%) were used as biopsy criteria. Serum samples were obtained before any prostatic manipulation and PSA in combination with fPSA% was evaluated in the laboratory of Urology, Medical University of Innsbruck on the same day.

PSA and fPSA concentration was assessed using the IMX assay (Abbott Laboratories, Abbott Park, IL, USA). From 1995 to 2000 ten systematic transrectal ultrasound-guided prostate biopsy cores were taken in a standard spatial distribution; from 2000 to 2012, an additional five elastography-directed biopsy cores were taken.

### Statistical analyses

We stratified our patient collective (n = 2225) according to age at biopsy into four different groups: ≤49 years, 50–59 years, 60–69 years and ≥70 years. All demographic, clinical, and histopathological characteristics were analyzed descriptively (absolute and relative frequency for qualitative data and mean, standard deviation [SD], median or interquartile range for quantitative data) and stratified by age groups.

Evaluating the effect of higher PSA limits than the currently used cut-offs we simulated different scenarios and calculated sensitivity and specificity for all of them (cut-off PSA values between 1.25 and 6 ng/mL (increments: 0.5 ng/mL) and fPSA% values between 15 and 21% (15%/18%/21%) for all four age groups separately). Applying current age-adjusted PSA references, sensitivity was at least 88% in all age groups. We therefore set this value as the lower limit of sensitivity for the new cut-off values and proposed the cut-off values with the highest specificity and predictive values among those with sensitivity ≥88% as new reference value. Sensitivity, specificity as well as positive and negative predictive values, including exact 95% confidence intervals (CIs) were calculated. Receiver operating characteristic (ROC) curves were constructed for PSA, stratified for fPSA% values ≤18% vs. >18% and ≤21% vs. >21%, respectively, to visually assess the discriminatory power for predicting PCa. Fisher’s exact test was performed to compare the number of biopsies to be done according to the “old” and “new” cut-off values. The number needed to screen to avoid one biopsy was calculated as the inverse of the relative biopsy frequency reduction. Statistical analyses were performed using the statistical software package SPSS, version 20.0 (SPPS Inc, Chicago, IL, USA). Figures were produced in R 3.1.1.

## Results

PSA, fPSA% and prostate volume at time of prostate biopsy as well as histology of biopsy specimens stratified according to age groups (≤49 years, 50–59 years, 60–69 years and ≥70 years) are shown in Table 1. Overall, PCa was diagnosed in 1218 men (54.7%). Out of this patient collective, 758 underwent radical prostatectomy at our department between 1995 and 2012. Analyzing the radical prostatectomy specimens we found that 35.8% of patients had a Gleason score (GS) ≥7 in the radical prostatectomy specimen (Table 2). Detailed histopathology data of radical prostatectomy specimens are summarized in Table 2.

**Table 1:** Clinical and demographic characteristics of the study population stratified by age.

	Group 1 (≤ 49 years)	Group 2 (50–59 years)	Group 3 (60–69 years)	Group 4 (≥ 70 years)	Total study population
Total patients (n, % of total study population)	178 (8.0%)	597 (26.8%)	962 (43.2%)	488 (21.9%)	2225 (100%)
Age (mean, SD, median, IQR)	45.4, 3.2, 46.0, [43.8,48.0]	55.3, 2.6, 55.0, [54.0,58.0]	64.5, 2.8, 65.0, [62.0,67.0]	74.2, 3.9, 73.0,[71.0,76.0]	62.6, 8.9, 63.0, [57.0,69.0]
PSA (ng/ml) (mean, SD, median, IQR)	4.2, 8.6, 2.8, [2.0,4.4]	7.7, 50.8, 4.5, [3.1,6.5]	9.9, 55.9, 5.6, [4.0,7.9]	28.6, 225.3, 7.1, [4.7,11.8]	12.9, 115.0, 5.2, [3.6,7.9]
fPSA (%) (mean, SD, median, IQR)	12.6, 6.0, 12.7, [9.0, 15.1]	14.4, 7.8, 13.9, [10.2,17.4]	16.1, 9.9, 14.9, [10.0,21.0]	16.9, 12.5, 15.0, [9.2,22.8]	15.5, 9.9, 14.3, [10.0,19.5]
Prostate volume (mean, SD, median, IQR)	28.2, 16.3, 26.0, [20.0, 35.0]	37.6, 20.2, 35.0, [26.0,45.0]	68.0, 292.2, 41.0, [30.0,61.0]	46.3, 32.6, 40.0, [26.0,61.5]	51.9,193.9,37.0,[26.0,55.0]
Benign biopsies (n, %)	117, 65.7%	302, 50.6%	438, 45.5%	150, 30.7%	1007, 45.3%
Malignant biopsies (n, %)	61, 34.3%	295, 49.4%	524, 54.5%	338, 69.3%	1218, 54.7%
Biopsy GS ≤6 (n, %)	46, 75.4%	202, 68.5%	291, 55.5%	133, 39.3%	672, 55.2%
Biopsy GS 7 (3+4) (n, %)	12, 19.7%	61, 20.7%	136, 26.0%	99, 29.3%	308, 25.3%
Biopsy GS 7 (4+3) (n, %)	2, 3.3%	13, 4.4%	27, 5.2%	24, 7.1%	66, 5.4%
Biopsy GS ≥8 (n, %)	1, 1.6%	17, 5.8%	65, 12.4%	77, 22.8%	160, 13.1%
Information on Biopsy GS missing	0, 0.0%	2, 0.7%	5, 1.0%	5, 1.5%	12, 1.0%

**Table 2:** Histopathological characteristics of radical prostatectomy specimens stratified by age.

	Group 1 (≤ 49 years)	Group 2 (50–59 years)	Group 3 (60–69 years)	Group 4 (≥ 70 years)	Total study population
Total patients (n, % of total study population)	54 (7.1%)	246 (32.5%)	371 (48.9%)	87 (11.5%)	758 (100%)
Age (mean, SD, median, IQR)	45.2, 3.5, 46.0, [43.0,48.0]	55.6, 2.6, 56.0, [54.0, 58.0]	64.3, 2.8, 64.0, [62.0, 67.0]	71.7, 1.8, 71.0, [71.0, 72.0]	61.0, 7.3, 62.0, [56.0, 66.0]
GS ≤6 (n, %)	43, 79.6%	176, 71.5%	217, 58.5%	43, 49.4%	479, 63.2%
GS 7 (3+4) (n, %)	8, 14.8%	49, 19.9%	99, 26.7%	24, 27.6%	180, 23.7%
GS 7 (4+3) (n, %)	2, 3.7%	12, 4.9%	22, 5.9%	5, 5.7%	41, 5.4%
GS ≥8 (n, %)	1, 1.9%	8, 3.3%	30, 8.1%	12, 13.8%	51, 6.7%
Information on GS missing	0, 0.0%	1, 0.4%	3, 0.8%	3, 3.4%	7, 0.9%
≤pT2c (n, %)	48, 88.9%	200, 81.3%	260, 70.1%	47, 54.0%	555, 73.2%
≥pT3a (n, %)	5, 9.3%	44, 17.9%	101, 27.2%	31, 35.6%	181, 23.9%
Information on pT missing	1, 1.9%	2, 0.8%	10, 2.7%	9, 10.3%	22, 2.9%
R0 (n, %)	42, 77.8%	188, 76.4%	257, 69.3%	49, 56.3%	536, 70.7%
R1 resection (n, %)	9, 16.7%	47, 19.1%	88, 23.7%	22, 25.3%	166, 21.9%
Information on R missing	3, 5.6%	11, 4.5%	26, 7.0%	16, 18.4%	56, 7.4%

**Fig 1:**
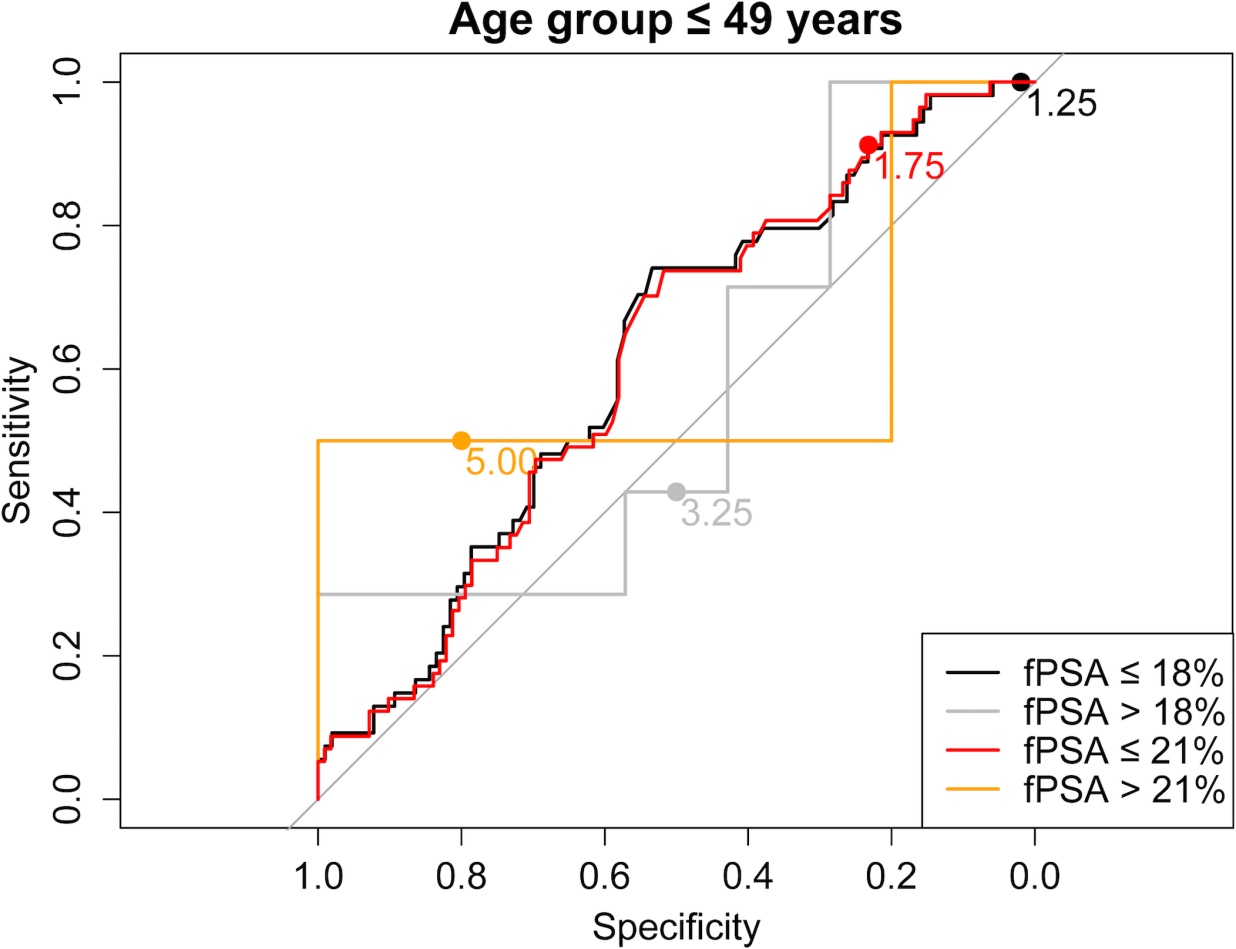
ROC curve for the age group ≤ 49 years stratified for fPSA values ≤18% vs. >18% and ≤21% vs. >21%, respectively.

**Fig 2:**
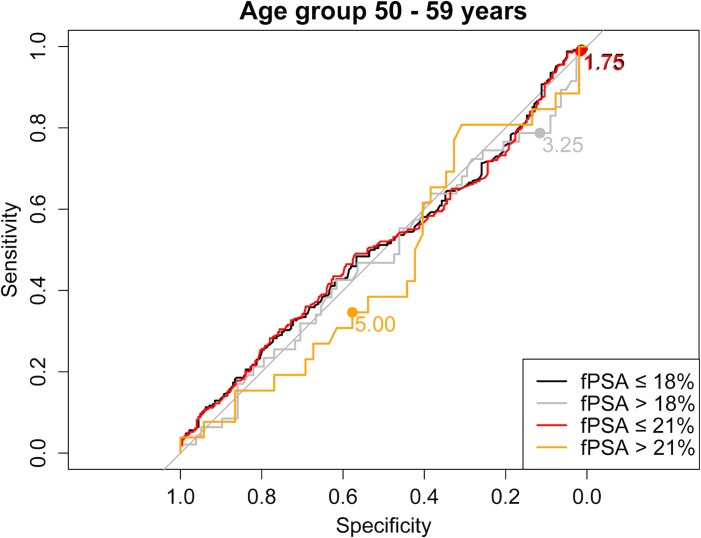
ROC curve for the age group ≤50–59 years stratified for fPSA values ≤18% vs. >18% and ≤21% vs. >21%, respectively.

**Fig 3:**
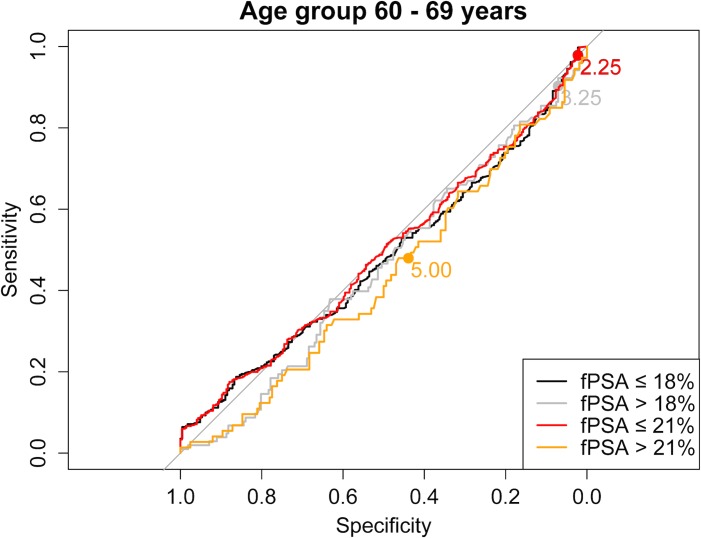
ROC curve for the age group 60–69 years stratified for fPSA values ≤18% vs. >18% and ≤21% vs. >21%, respectively.

**Fig 4:**
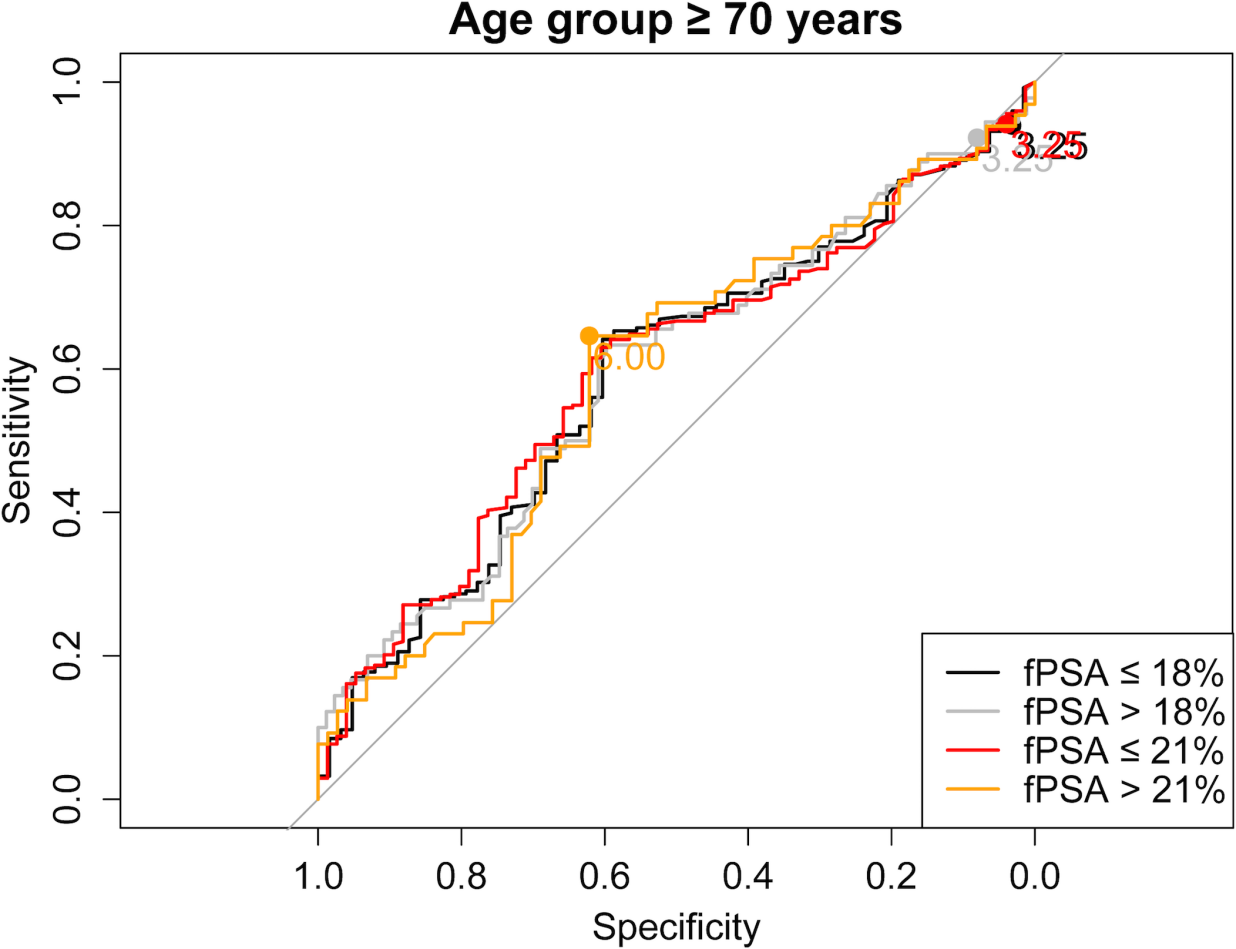
ROC curve for the age group ≥ 70 years stratified for fPSA values ≤18% vs. >18% and ≤21% vs. >21%, respectively.

As a next step we evaluated the use of higher than the current PSA cut-offs with the aim of reducing the number of biopsies, without a relevant loss in PCa prediction. S1 Table shows sensitivity and specificity of all different PSA and fPSA% cut-off combinations for each age group separately. Moreover, ROC curves stratified for fPSA values ≤18% vs. >18% and ≤21% vs. >21%, respectively, are shown in Figs 1–4 for the different age groups.

Based on these findings we propose the following “new” PSA cut-offs in combination with fPSA% ≤21%: 1.75 ng/ml for men ≤49 years and 50–59 years, 2.25 ng/ml for men aged 60–69 years and 3.25 ng/ml for men ≥70 years (Table 3).

**Table 3:** Proposed “new” PSA cut offs stratified by age.

Age group	Cut-off for PSA with fPSA ≤ 21%	Cut-off for PSA with fPSA *>* 21%
≤ 49 years	1.75 ng/ml	5 ng/ml
50–59 years	1.75 ng/ml	5 ng/ml
60–69 years	2.25 ng/ml	5 ng/ml
≥ 70 years	3.25 ng/ml	6 ng/ml

Comparison of specificity, sensitivity as well the positive and negative predictive value of both “old” and “new” PSA cut-offs reveal comparable positive and negative prediction values of both “old” and “new” PSA cut-offs as shown in Table 4. The “new” cut-off values achieved a similarly high sensitivity for detecting significant tumors compared to the current ones (current sensitivities in Table 5, new sensitivities in Table 6). All significant tumors were recognized in all age groups by both cut-off schemas (Table 6).

**Table 4:** Comparison of “new” and “old” PSA cut offs concerning specificity (spec), sensitivity (sens) as well as positive (pos pred) and negative predictive (neg pred) values stratified by age.

Age group	Sens old cut-offs	Sens new cut-offs	Spec old cut-offs	Spec new cut-offs	Pos pred old cut-offs	Pos pred new cut-offs	Neg pred old cut-offs	Neg pred new cut-offs
≤ 49 years	93.4%	88.5%	7.7%	25.6%	34.5%	38.3%	69.2%	81.1%
50–59 years	96.9%	93.2%	4.0%	11.3%	49.4%	50.6%	50.0%	63.0%
60–69 years	96.4%	90.8%	4.6%	17.8%	54.7%	56.9%	51.3%	61.9%
≥ 70 years	93.5%	88.5%	6.0%	32.7%	69.1%	74.8%	29.0%	55.7%

**Table 5:** Recognized tumors (%) biopsied according to “old” PSA cut offs stratified by age.

Age group	GS6	GS 3+4	GS 4+3	GS 8	GS 9	10	≤pT2c	≥pT3a
≤ 49 years	95.1%	91.7%	100%	100%	No data	No data	97.9%	100%
50–59 years	96.7%	100%	100%	100%	100%	100%	98%	100%
60–69 years	97.3%	97.1%	71.7%	97.7%	94.4%	100%	98.5%	97%
≥ 70 years	96%	100%	100%	100%	95.6%	100%	97.9%	96.8%

**Table 6:** Recognized tumors (%) biopsied according to “new” PSA cut offs stratified by age.

Age group	GS6	GS 3+4	GS 4+3	GS 8	GS 9	GS 10	≤pT2c	≥pT3a
≤ 49 years	97.6%	91.7%	100%	100%	No data	No data	97.9%	100%
50–59 years	96.7%	100%	100%	100%	100%	100%	98%	100%
60–69 years	97.3%	97.1%	71.7%	97.7%	94.4%	100%	98.5%	97%
≥ 70 years	96%	100%	100%	100%	95.6%	100%	97.9%	96.8%

Using the “new” PSA cut-offs significantly reduced the number of prostate biopsies among all age groups (Table 7). Overall, one biopsy is avoided in 13 to 14 men (number needed to screen = 13.3, reduction of biopsies = 7.5%) when decision regarding biopsy is done according to the “new” cut-off values instead of the “old” ones. This avoids biopsy in 7.5% of men. For the different age groups the number needed to screen to avoid one biopsy varied between 9.2 (≤49 years) and 17.4 (50–59 years) (Table 7).

**Table 7:** Reduction of number of biopsies with the “new” PSA cut offs stratified by age.

Age group	Difference in % (95% CI)	Number Needed To screen (95% CI)	P Value
≤ 49 years	10.9% (2.4%-19.4%)	9.2 (5.1–42.2)	**0.017**
50–59 years	5.7% (1.3%-10.1%)	17.4 (9.9–74.8)	**0.013**
60–69 years	7.2% (3.7%-10.8%)	13.8 (9.3–26.9)	**<0.001**
≥ 70 years	9.0% (3.9%-14.1%)	11.1 (7.1–25.9)	**0.001**
Total study population	7.5% (5.1%-9.9%)	13.3 (10.1–19.3)	**<0.001**

## Discussion

The present study evaluated the biopsy PSA cut-off values used since 1995 in the Tyrol study with the aim to reduce overdiagnosis and consequently overtreatment of PCa as the current PSA based biopsy criteria are more restrictive compared to other PCa early detection programs. In summary our results propose “new” age-adjusted PSA cut-offs with higher PSA limits, which reduce the number of biopsies without compromizing significant PCa detection.

The introduction of PSA screening has modified the epidemiology of the disease leading to stage migration towards mostly curative forms of the disease [2,15]. Several large studies have shown that men who are not participating in PCa early detection programs have more aggressive forms of cancers compared to those who were participating in PCa early detection programs. In Tyrol PSA screening was introduced already in the early nineties thereby significantly reducing PCa mortality compared to the other parts of Austria [11,13]. 54.7% of our study population was diagnosed with PCa, among them 55.2% were biopsy GS 6 cancers. Thus, a large proportion of patients were diagnosed and consequently treated with low risk forms of the disease.

On the other hand, analysing the corresponding radical prostatectomy specimens of biopsy cores we found that a significant number of cancers were upgraded in the final histology, thereby leading to significant forms of carcinomas which would need further therapy. These findings are in line with previous findings by us and other groups that 52.4% of patients are upgraded in the histology of the radical prostatectomy specimen [16,17]. Moreover, recently Bill-Axelson et al showed in a large cohort of low risk PCa patients that after 12.8 years radical prostatectomy was associated with a reduction in the rate of death from PCa compared to watchful waiting [18]. These data would strongly support the concept of detecting and diagnosing also low risk carcinomas.

8% of our patient collective included men ≤ 49 years, among them more than 20% had ≥ GS 7 carcinoma in the histology of the radical prostatectomy specimen. This finding underlines the concept of PSA measurement starting at early age. In the age group ≤ 49 years, in the present study the median PSA level was 2.8 ng/ml which is much higher than in most published series, where it has been shown that the median PSA level ranges between 0.4 and 0.7 ng/ml in this age group [19]. Our data are in line with another study that described significant PCa in 24% of a < 50 years study population [20]. However, a Spanish retrospective analysis of 69 patients under 50 years of age diagnosed with PCa found that 60% had metastatic tumors at initial diagnosis. In this patient collective, mean survival was 16.1 months (1–84), and all patients died due to disease progression [21].

The present study demonstrates that both “old” and “new” PSA cut-offs recognize PCa with a high probability (97–100%). However, the “new” PSA cut-offs have the advantage that they significantly reduce the number of biopsies in all age groups. This means that we detect all significant tumors despite of a reduction of biopsies. Thereby it has also to be considered that PSA in combination with fPSA is not the only biopsy indication as digital rectal examination, PSA velocities or PSA isoforms are also involved in the decision. Also imaging methods like real-time elastography or multiparemetic MRI depict PCa with high sensitivity in our patient collective [22–24].

Using “new” PSA cut-offs a significant number of prostate biopsies can be reduced. For the different age groups the number needed to screen to avoid one biopsy varied between 9.2 (≤49 years) and 17.4 (50–59 years) with the most biopsy savings in the youngest age group. Thus, especially in young patients where prostatitis is the most common cause of PSA elevation, unnecessary biopsies can be avoided using the higher PSA cut-offs. In addition, biopsy reduction is associated with cost–and personal savings and reduces psychological stress factors in patients. Moreover, it has to be considered that surgical events always might have intra-or postoperative side effects.

For the future it will be interesting to analyze in our patient cohort if the incidence of low risk and thus insignificant tumors reduced since the introduction of the “new” PSA cut-offs.

In general, 70–90% of total PSA is present as a complexed form in serum associated with numerous different endogenous protease inhibitors, which prevent damage by the protease activity of PSA. However, 10 to 30% of PSA is present in an uncomplexed form, so called, free PSA (fPSA). The use of percentage of free PSA (fPSA%) for PCa prediction has successfully improved the accuracy of cancer detection compared to PSA measurement alone [25,26]. Although there is general agreement that higher PSA values are associated with a better prognosis, the exact amount of the value has not yet been determined.

In the present study we were able to show, that an fPSA% change from 18% to 21% regarding biopsy cut-offs for PSA with fPSA >21% (Table 3) leading to a significant increase in PCa detection specificity and sensitivity (12).

Our study has several limitations like the two different biopsy techniques (10 cores versus 15 cores) as well as the absence of reference pathologies for diagnosis of histo-pathological specimens. Also loss of follow up of patients who would have developed PCa as well as the possibility of false negative biopsies has to be considered. In addition, compared to other screening studies like the ERSPC or der PLCO study, the present investigation includes a small patient collective especially in the age group ≤ 49 years (n = 178). Moreover, there is need to validate the proposed “new” PSA cut-offs in the daily clinical routine as well as in prospective multicenter studies.

In summary, the proposed PSA cut-offs are able to detect PCa with a high sensitivity and specificity, without missing significant forms of the disease. The “new” PSA cut-offs is one step towards a smarter strategy in the Tyrol PCa Early Detection Program.

## Supporting Information

S1 Table Sensitivity and specificity of different cut-off value scenarios, stratified by age categories. (DOCX)
