# Supplementary material for: Age-Adjusted PSA Levels in Prostate Cancer Prediction: Updated Results of the Tyrol Prostate Cancer Early Detection Program
Source: PLoS One. 2015 Jul 28;10(7):e0134134. doi: 10.1371/journal.pone.0134134 (PMC4517762; doi:10.1371/journal.pone.0134134)
Supplement: S1 Table — (DOCX) [file pone.0134134.s001.docx]

Supplementary material

Table Supplementary 1: Sensitivity and specificity of different cut-off value scenarios, stratified by age categories

| **Age group** | **Sensitivity** | **Specificity** | **Cut-off value fPSA** | **Cut-off value PSA^a^** |
| --- | --- | --- | --- | --- |
| ≤ 49 years | 83.6% | 19.7% | 15% | 1.25 / 3.25 |
|  | 82.0% | 22.2% | 15% | 1.25 / 4.00 |
|  | 82.0% | 25.6% | 15% | 1.25 / 5.00 |
|  | 77.0% | 33.3% | 15% | 1.75 / 3.25 |
|  | 75.4% | 35.9% | 15% | 1.75 / 4.00 |
|  | 75.4% | 39.3% | 15% | 1.75 / 5.00 |
|  | 67.2% | 47.9% | 15% | 2.25 / 3.25 |
|  | 65.6% | 50.4% | 15% | 2.25 / 4.00 |
|  | 65.6% | 53.8% | 15% | 2.25 / 5.00 |
|  | 60.7% | 57.3% | 15% | 2.75 / 3.25 |
|  | 59.0% | 59.8% | 15% | 2.75 / 4.00 |
|  | 59.0% | 63.2% | 15% | 2.75 / 5.00 |
|  | 93.4%^b^ | 7.7%^b^ | 18%^b^ | 1.25 / 3.25^b^ |
|  | 93.4% | 7.7% | 18% | 1.25 / 4.00 |
|  | 91.8% | 11.1% | 18% | 1.25 / 5.00 |
|  | 85.2% | 26.5% | 18% | 1.75 / 3.25 |
|  | 83.6% | 28.2% | 18% | 1.75 / 4.00 |
|  | 83.6% | 29.9% | 18% | 1.75 / 5.00 |
|  | 73.8% | 41.9% | 18% | 2.25 / 3.25 |
|  | 72.1% | 43.6% | 18% | 2.25 / 4.00 |
|  | 72.1% | 45.3% | 18% | 2.25 / 5.00 |
|  | 65.6% | 55.6% | 18% | 2.75 / 3.25 |
|  | 63.9% | 57.3% | 18% | 2.75 / 4.00 |
|  | 63.9% | 59.0% | 18% | 2.75 / 5.00 |
|  | 96.7% | 2.6% | 21% | 1.25 / 3.25 |
|  | 96.7% | 3.4% | 21% | 1.25 / 4.00 |
|  | 96.7% | 5.1% | 21% | 1.25 / 5.00 |
|  | 88.5% | 23.1% | 21% | 1.75 / 3.25 |
|  | 88.5% | 23.9% | 21% | 1.75 / 4.00 |
|  | 88.5%^c^ | 25.6%^c^ | 21%^c^ | 1.75 / 5.00^c^ |
|  | 75.4% | 39.3% | 21% | 2.25 / 3.25 |
|  | 75.4% | 40.2% | 21% | 2.25 / 4.00 |
|  | 75.4% | 41.9% | 21% | 2.25 / 5.00 |
|  | 67.2% | 53.8% | 21% | 2.75 / 3.25 |
|  | 67.2% | 54.7% | 21% | 2.75 / 4.00 |
|  | 67.2% | 56.4% | 21% | 2.75 / 5.00 |
| 50-59 years | 86.1% | 9.3% | 15% | 1.75 / 3.25 |
|  | 81.0% | 17.2% | 15% | 1.75 / 4.00 |
|  | 75.6% | 28.1% | 15% | 1.75 / 5.00 |
|  | 81.4% | 14.2% | 15% | 2.25 / 3.25 |
|  | 76.3% | 22.2% | 15% | 2.25 / 4.00 |
|  | 70.8% | 33.1% | 15% | 2.25 / 5.00 |
|  | 75.3% | 18.9% | 15% | 2.75 / 3.25 |
|  | 70.2% | 26.8% | 15% | 2.75 / 4.00 |
|  | 64.7% | 37.7% | 15% | 2.75 / 5.00 |
|  | 67.8% | 24.8% | 15% | 3.25 / 3.25 |
|  | 62.7% | 32.8% | 15% | 3.25 / 4.00 |
|  | 57.3% | 43.7% | 15% | 3.25 / 5.00 |
|  | 96.9%^b^ | 3.3%^b^ | 18%^b^ | 1.75 / 3.25^b^ |
|  | 96.9% | 3.3% | 18% | 1.75 / 4.00 |
|  | 90.2% | 16.9% | 18% | 1.75 / 5.00 |
|  | 88.8% | 10.6% | 18% | 2.25 / 3.25 |
|  | 86.4% | 16.2% | 18% | 2.25 / 4.00 |
|  | 83.1% | 23.5% | 18% | 2.25 / 5.00 |
|  | 78.0% | 16.9% | 18% | 2.75 / 3.25 |
|  | 75.6% | 22.5% | 18% | 2.75 / 4.00 |
|  | 72.2% | 29.8% | 18% | 2.75 / 5.00 |
|  | 67.8% | 24.8% | 18% | 3.25 / 3.25 |
|  | 65.4% | 30.5% | 18% | 3.25 / 4.00 |
|  | 62.0% | 37.7% | 18% | 3.25 / 5.00 |
|  | 97.6% | 3.0% | 21% | 1.75 / 3.25 |
|  | 95.9% | 7.3% | 21% | 1.75 / 4.00 |
|  | 93.2%^c^ | 11.3%^c^ | 21%^c^ | 1.75 / 5.00^c^ |
|  | 90.5% | 9.6% | 21% | 2.25 / 3.25 |
|  | 88.8% | 13.9% | 21% | 2.25 / 4.00 |
|  | 86.1% | 17.9% | 21% | 2.25 / 5.00 |
|  | 79.0% | 16.2% | 21% | 2.75 / 3.25 |
|  | 77.3% | 20.5% | 21% | 2.75 / 4.00 |
|  | 74.6% | 24.5% | 21% | 2.75 / 5.00 |
|  | 67.8% | 24.8% | 21% | 3.25 / 3.25 |
|  | 66.1% | 29.1% | 21% | 3.25 / 4.00 |
|  | 63.4% | 33.1% | 21% | 3.25 / 5.00 |
| 60-69 years | 92.7% | 6.6% | 15% | 2.25 / 3.25 |
|  | 87.0% | 15.8% | 15% | 2.25 / 4.00 |
|  | 80.5% | 28.5% | 15% | 2.25 / 5.00 |
|  | 88.0% | 8.2% | 15% | 2.75 / 3.25 |
|  | 82.3% | 17.4% | 15% | 2.75 / 4.00 |
|  | 75.8% | 30.1% | 15% | 2.75 / 5.00 |
|  | 84.7% | 9.6% | 15% | 3.25 / 3.25 |
|  | 79.0% | 18.7% | 15% | 3.25 / 4.00 |
|  | 72.5% | 31.5% | 15% | 3.25 / 5.00 |
|  | 74.0% | 21.7% | 15% | 4.00 / 4.00 |
|  | 67.6% | 34.5% | 15% | 4.00 / 5.00 |
|  | 96.4%^b^ | 4.6%^b^ | 18%^b^ | 2.25 / 3.25^b^ |
|  | 96.4% | 4.6% | 18% | 2.25 / 4.00 |
|  | 88.7% | 23.1% | 18% | 2.25 / 5.00 |
|  | 90.3% | 7.1% | 18% | 2.75 / 3.25 |
|  | 86.6% | 15.3% | 18% | 2.75 / 4.00 |
|  | 82.6% | 25.6% | 18% | 2.75 / 5.00 |
|  | 84.7% | 9.6% | 18% | 3.25 / 3.25 |
|  | 81.1% | 17.8% | 18% | 3.25 / 4.00 |
|  | 77.1% | 28.1% | 18% | 3.25 / 5.00 |
|  | 74.0% | 21.7% | 18% | 4.00 / 4.00 |
|  | 70.0% | 32.0% | 18% | 4.00 / 5.00 |
|  | 96.8% | 3.4% | 21% | 2.25 / 3.25 |
|  | 94.1% | 9.4% | 21% | 2.25 / 4.00 |
|  | 90.8%^c^ | 17.8%^c^ | 21%^c^ | 2.25 / 5.00^c^ |
|  | 90.5% | 6.2% | 21% | 2.75 / 3.25 |
|  | 87.8% | 12.1% | 21% | 2.75 / 4.00 |
|  | 84.5% | 20.5% | 21% | 2.75 / 5.00 |
|  | 84.7% | 9.6% | 21% | 3.25 / 3.25 |
|  | 82.1% | 15.5% | 21% | 3.25 / 4.00 |
|  | 78.8% | 24.0% | 21% | 3.25 / 5.00 |
|  | 74.0% | 21.7% | 21% | 4.00 / 4.00 |
|  | 70.8% | 30.1% | 21% | 4.00 / 5.00 |
| ≥ 70 years | 93.5% | 6.0% | 15% | 3.25 / 3.25 |
|  | 90.5% | 14.0% | 15% | 3.25 / 4.00 |
|  | 84.0% | 27.3% | 15% | 3.25 / 5.00 |
|  | 80.8% | 39.3% | 15% | 3.25 / 6.00 |
|  | 87.6% | 16.7% | 15% | 4.00 / 4.00 |
|  | 81.1% | 30.0% | 15% | 4.00 / 5.00 |
|  | 77.8% | 42.0% | 15% | 4.00 / 6.00 |
|  | 73.4% | 36.7% | 15% | 5.00 / 5.00 |
|  | 70.1% | 48.7% | 15% | 5.00 / 6.00 |
|  | 66.3% | 54.7% | 15% | 6.00 / 6.00 |
|  | 93.5%^b^ | 6.0%^b^ | 18%^b^ | 3.25 / 3.25^b^ |
|  | 93.5% | 6.0% | 18% | 3.25 / 4.00 |
|  | 87.3% | 24.7% | 18% | 3.25 / 5.00 |
|  | 85.8% | 36.0% | 18% | 3.25 / 6.00 |
|  | 87.6% | 16.7% | 18% | 4.00 / 4.00 |
|  | 83.1% | 28.7% | 18% | 4.00 / 5.00 |
|  | 81.7% | 40.0% | 18% | 4.00 / 6.00 |
|  | 73.4% | 36.7% | 18% | 5.00 / 5.00 |
|  | 71.9% | 48.0% | 18% | 5.00 / 6.00 |
|  | 66.3% | 54.7% | 18% | 6.00 / 6.00 |
|  | 93.5% | 6.0% | 21% | 3.25 / 3.25 |
|  | 92.0% | 12.7% | 21% | 3.25 / 4.00 |
|  | 89.6% | 23.3% | 21% | 3.25 / 5.00 |
|  | 88.5% ^c^ | 32.7%^c^ | 21%^c^ | 3.25 / 6.00^c^ |
|  | 87.6% | 16.7% | 21% | 4.00 / 4.00 |
|  | 85.2% | 27.3% | 21% | 4.00 / 5.00 |
|  | 84.0% | 36.7% | 21% | 4.00 / 6.00 |
|  | 73.4% | 36.7% | 21% | 5.00 / 5.00 |
|  | 72.2% | 46.0% | 21% | 5.00 / 6.00 |
|  | 66.3% | 54.7% | 21% | 6.00 / 6.00 |

^a^: The first PSA cut-off value is used if fPSA is equal or smaller than indicated in the fPSA cut-off value column, the second PSA cut-off value is used if fPSA is larger than indicated in the fPSA cut-off value column; ^b^: “Old” cut-off values; ^c^: “New” cut-off values
